# Esophageal Trachealization: A Feature of Eosinophilic Esophagitis

**DOI:** 10.4103/1319-3767.54747

**Published:** 2009-07

**Authors:** Abdulrahman A. Al-Hussaini, Toufic Semaan, Imad A. El Hag

**Affiliations:** 1 Department of Medicine, Children's Hospital, King Fahad Medical City, King Saud Medical Complex, Riyadh, Saudi Arabia; 2 Department of Pathology, Children's Hospital, King Fahad Medical City, King Saud Medical Complex, Riyadh, Saudi Arabia

**Keywords:** Eosinophilic esophagitis, esophageal rings, fluticasone

## Abstract

Eosinophilic esophagitis (EE) is an inflammatory condition characterized by intense eosinophilic infiltration of the esophagus. EE is frequently misdiagnosed as gastroesophageal reflux disease. Here, we present a child with EE and a characteristic endoscopic finding, “ringed esophagus”. An 11-year-old Saudi boy presented with dysphagia for 1 year. He had experienced an intermittent sensation of solid food sticking in his chest, which was relieved by drinking liquids. A barium swallow excluded anatomical causes of dysphagia, but revealed multiple-ringed esophagus. Endoscopy showed a furrowing and trachealizing appearance of the entire esophagus. Hisologically, extensive eosinophilic infiltration was a feature in biopsies obtained from the esophagus. The child responded well to a 2-month course of inhaled fluticasone. Symptoms recurred 3 months after discontinuation of therapy, which necessitated resumption of inhaled fluticasone. The endoscopic appearance of multiple esophageal rings should raise suspicion of EE and be confirmed by esophageal biopsies.

Eosinophilic esophagitis (EE) is an inflammatory condition characterized by extensive infiltration of eosinophils into the esophagus. Although the symptoms are similar to those of gastroesophageal reflux disease (GERD), patients with EE often experience mild or no response to acid suppression and other forms of antireflux therapy. Reflux esophagitis may present an endoscopic picture similar to EE with erythema, loss of vascular pattern, and edema. However, ulceration is not a feature of EE. In contrast, the presence of an esophageal ring throughout the entire length of the esophagus is a rare but characteristic endoscopic finding in EE. We present a child with multiple esophageal rings in a biopsy-proven EE.

## CASE REPORT

A 9-year-old Saudi boy presented with dysphagia and recurrent food sticking after eating solid food for 1 year. He had repeated choking episodes in the past, which were relieved by drinking fluids or coughing up food particles. Because of his reluctance to eat solids, he lost some weight. There was no history of abdominal pain, regurgitation, vomiting, atopy, drug, or caustic ingestion. Because of suspicion of GERD, he received oral omeprazole with some improvement. The past medical and surgical history is unremarkable.

Physical examination revealed a thin boy with both weight (21 kg) and height (122 cm) just below the fifth percentile for age, normal vital signs, no lymphadenopathy, no skin rash, and normal musculoskeletal examination. The abdomen was soft and lax, with no hepatosplenomegaly. Other systemic examination was unremarkable.

**Figure 1:**
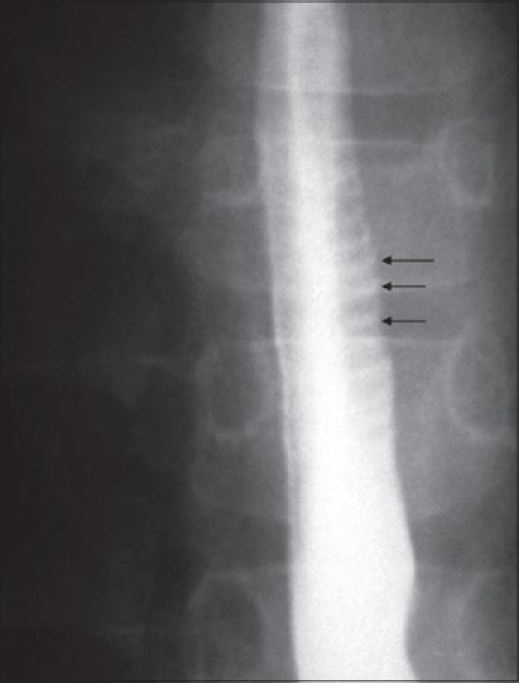
Barium swallow study reveals multiple esophageal rings (arrows)

**Figure 2:**
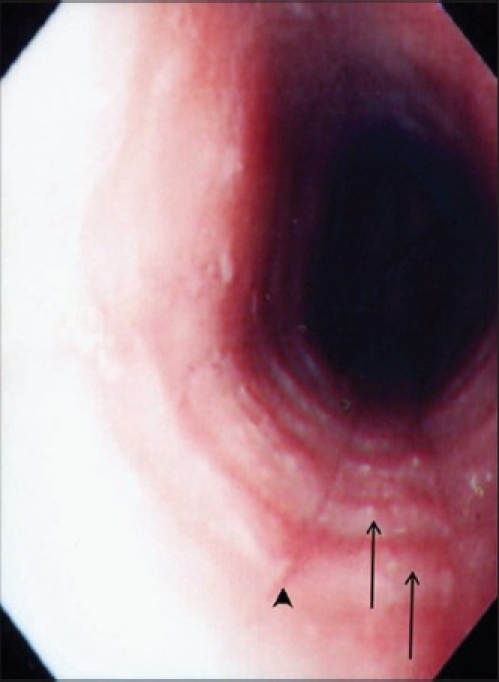
Upper endoscopy revels multiple esophageal rings (arrows) and furrowings (arrow head)

**Figure 3:**
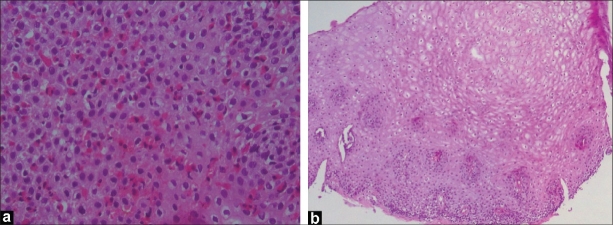
(a) Esophageal biopsy with eosinophilic infiltrate >20 eosinophils/HPF (×100); (b) Normal esophageal biopsy 6 weeks after treatment with fluticasone inhaler (×40)

Initial laboratory investigations showed hemoglobin of 12 g/L, white blood cell count of 5.4 × 10^9^, and normal differential count, platelets 334 × 10^6^, erythrocyte sedimentation rate 10mm/h, serum total protein 68 gm/L, serum albumin 38 gm/L, alanine aminotransferase 34 IU/L, aspartate aminotransferase 41 IU/L, alkaline phosphatase 350 IU/L, total serum bilirubin 10 *μ*g/L, and normal urea, creatinine, and electrolytes. There were no ova or parasites on stool analysis. Barium swallow study to evaluate the anatomic causes of dysphagia revealed a ringed esophagus [Figure 1] and no evidence of stricture. Endoscopic examination revealed multiple rings throughout the esophagus [Figure 2]. The esophageal mucosa was edematous, friable and pale, with loss of vascular pattern. Furrowing of the esophageal mucosa was prominent [Figure 2]. Multiple biopsies were obtained from the lower, middle, and upper esophagus. The mucosa of the stomach and the duodenum looked normal. Histological examination of biopsies from different parts of the esophagus showed extensive infiltration of the mucosa with eosinophils (>20 eosinophils/HPF) [Figure 3a].

Therefore, a trial of inhaled fluticasone 375 *μ*g twice a day (each puff (125 *μ*g) is swallowed rather than inhaled) was initiated for 1 month in adjunct with omeprazole (1mg/kg/day). This led to a complete resolution of the symptoms and weight gain. The dose of fluticasone was weaned to 250 *μ*g twice a day over the next month before stoppage. Repeat of endoscopy and esophageal biopsies showed disappearance of esophageal rings and histological recovery [Figure 3b].

Dysphagia recurred 3 months later and, although endoscopy revealed no esophageal rings, biopsies from the esophagus were extensively infiltrated with eosinophils. Another course of fluticasone inhaler was initiated at 375 *μ*g twice a day with good symptomatic recovery in 1 week. The dose was weaned over 2 months and maintained at 125 *μ*g twice a day for 1 year. On follow-up, the child is still asymptomatic 1 year after initiation of therapy.

## DISCUSSION

The etiology of EE is not known. An increased emphasis has been placed on the role of food allergy. A type 4 cell-mediated reaction is most likely to be involved.[1]

Typical symptoms include vomiting, regurgitation, epigastric pain, and dysphagia. Therefore, gastroenterologists often make a presumptive diagnosis of GERD and treat with antireflux therapy. The delay of 1 year in diagnosing EE in this patient may reflect this fact and the under recognition of this important clinical entity. Another common presentation of EE in children is esophageal food impaction that necessitates endoscopic retrieval. In such sitting, EE can be readily missed if the endoscopist fails to recognize the characteristic endoscopic findings of EE and when biopsies from the mid- and upper esophagus are not performed.

A definitive diagnosis of EE is made by identifying an isolated eosinophilic infiltration in the esophagus of patients who have reflux-like symptoms and who are refractory to acid blockade.[2] Multiple esophageal rings constitute a rare endoscopic finding. In the literature, a handful of cases have been reported in children and adults.[3–9] Barium swallow is usually performed as an initial study in the work-up of dysphagia to evaluate for the presence of stricture or findings suggestive of achalasia. In addition, a ringed esophagus is an important rare radiological finding that should be looked for in patients with dysphagia. These rings tend to disappear after appropriate treatment.[8]

Histologically, an eosinophil count >20/HPF, intra-epithelial eosinophils, eosinophilic micro-abscesses in the lamina propria, and presence of eosinophilia in biopsies from the mid- and upper esophagus are more characteristic of EE rather than reflux esophagitis.[2] Histological healing can be achieved by medical therapy, as demonstrated in our case.

Therapeutic options for EE include a strict elimination diet (e.g., amino acid-based formula),[10] oral or inhaled corticosteroids,[11–13] and mast cell stabilizer. Recurrence of EE within a year of discontinuation of corticosteroids is high (up to 90%).[11] The symptomatic improvement that was achieved by the use of omeprazole could be explained by the reduced exposure of an already diseased esophagus to acid.

One limitation of this report is that no skin patch allergy testing was performed in order to identify and eliminate possibly implicated food allergens.

In conclusion, the endoscopic appearance of multiple esophageal rings and furrowing should raise suspicion of EE and prompt initiation of appropriate therapy. Biopsy of the lower, middle, and upper esophagus is required on suspicion of EE on endoscopy.
